# Effectiveness of an Embedded Infectious Disease Screening, Treatment, and Prevention Intervention Within an Inpatient Substance Use Treatment Program

**DOI:** 10.1093/ofid/ofaf403

**Published:** 2025-07-24

**Authors:** Kelly E Dyer, Rebecca Russell, Rayek Nafiz, Angela Burdick-McPhee, Jean O’Neal, Tanajsia Mason, Danica Kuncio, Hannah Zellman, Margaret Lowenstein, Nancy Aitcheson, Vincent Lo Re, Jessie Torgersen

**Affiliations:** University of Pennsylvania Health System, Division of Infectious Diseases, Philadelphia, PA, USA; University of Pennsylvania Health System, Division of General Internal Medicine, Philadelphia, PA, USA; University of Pennsylvania Health System, Division of Psychiatry, Philadelphia, PA, USA; University of Pennsylvania Perelman School of Medicine, Philadelphia, PA, USA; University of Pennsylvania Health System, Division of Psychiatry, Philadelphia, PA, USA; University of Pennsylvania Health System, Division of Infectious Diseases, Philadelphia, PA, USA; Philadelphia Department of Public Health, Philadelphia, PA, USA; Philadelphia Department of Public Health, Philadelphia, PA, USA; Health Federation of Philadelphia, PA, USA; University of Pennsylvania Health System, Division of General Internal Medicine, Philadelphia, PA, USA; University of Pennsylvania Perelman School of Medicine, Philadelphia, PA, USA; University of Pennsylvania Perelman School of Medicine, Philadelphia, PA, USA; University of Pennsylvania Health System, Division of Infectious Diseases, Philadelphia, PA, USA; University of Pennsylvania Perelman School of Medicine, Philadelphia, PA, USA; Department of Biostatistics, Epidemiology, and Informatics, Center for Clinical Epidemiology and Biostatistics, Center for Real-world Effectiveness and Safety of Therapeutics, Perelman School of Medicine, University of Pennsylvania, Philadelphia, Pennsylvania, USA; University of Pennsylvania Health System, Division of Infectious Diseases, Philadelphia, PA, USA; University of Pennsylvania Perelman School of Medicine, Philadelphia, PA, USA; Department of Biostatistics, Epidemiology, and Informatics, Center for Clinical Epidemiology and Biostatistics, Center for Real-world Effectiveness and Safety of Therapeutics, Perelman School of Medicine, University of Pennsylvania, Philadelphia, Pennsylvania, USA

**Keywords:** harm reduction, infectious diseases, substance use disorder, viral hepatitis, vaccination uptake

## Abstract

**Background:**

People with substance use disorders (SUDs) are at higher risk for infectious diseases (IDs). Co-locating ID screening services within inpatient SUD treatment programs may decrease barriers to care; however, the impact of such screening has not been evaluated.

**Methods:**

We conducted an effectiveness study evaluating comprehensive ID screening within an 18-bed inpatient SUD program. During usual care (September 2021–June 2022), ID screening was completed at the discretion of the admitting psychiatrist. During the intervention (September 2022–June 2023), an ID-trained nurse met with patients to support completion of screening for HIV, viral hepatitis (hepatitis A [HAV], B [HBV], and C [HCV]), latent tuberculosis [LTBI], and sexually transmitted infections [STI]. Hepatitis vaccinations, HIV preexposure prophylaxis, and/or ID treatments were offered during admission.

**Results:**

Demographics were similar between the groups (n = 261, usual care; n = 207, intervention). Screening for ≥1 ID increased significantly during the intervention (60.2% vs 90.8%, *P* < .001), with the greatest increases in HAV (6.1% vs 90.3%, *P* < .001), HBV (8.8% vs 91.3%, *P* < .001), and LTBI (1.9% vs 67.8%, *P* < .001). HAV and HBV vaccinations increased from 0% to 58% and 71%, respectively. HCV viremia was identified in 15 usual care and 19 intervention patients, of whom 0% and 36.8% initiated direct-acting antiviral therapy within 2 weeks of testing. STIs were identified in 9.2% and 13.5% (*P* = .09) of the patients in the usual care and intervention groups.

**Conclusions:**

Comprehensive ID screening within inpatient SUD programs can increase uptake of testing and facilitate low-barrier delivery of preventive and therapeutic treatment.

## Teaser

Embedded infectious disease screening in an inpatient substance use disorder treatment program significantly increases screening frequency and can facilitate timely initiation of treatment and preventive measures, improving health care delivery for patients.

## Graphical Abstract

**Graphical Abstract:**
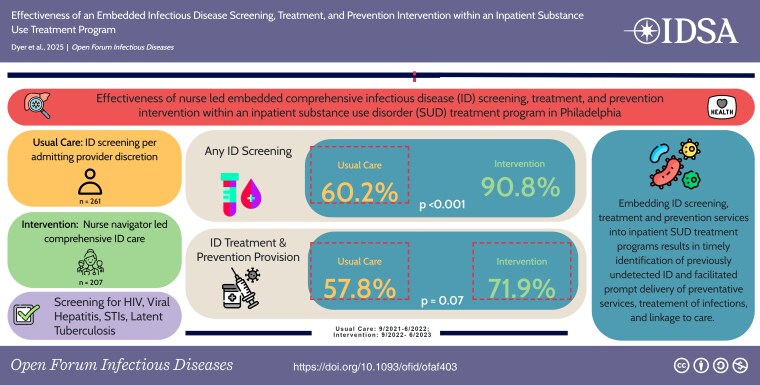
This graphical abstract is also available at Tidbit: https://tidbitapp.io/tidbits/real-world-effectiveness-of-an-embedded-infectious-disease-screening-treatment-and-prevention-program-within-an-inpatient-substance-use-treatment-program

The intersecting challenge of substance use disorders (SUDs) and infectious diseases (IDs) has emerged as a complex public health issue of paramount importance. People with SUD are at an elevated risk for HIV [1, 2], viral hepatitis (hepatitis A virus [HAV], hepatitis B virus [HBV], and hepatitis C virus [HCV]) [3–5], latent tuberculosis infection (LTBI) [6], and sexually transmitted infections (STIs) [7–9] relative to people without SUD. While these infections are preventable [10, 11] or treatable, many people are unaware of their infection status [12, 13], resulting in ongoing community transmission [13], delayed treatment initiation, and missed prevention opportunities.

Guidelines have called for regular screening for ID among people with SUD [14, 15]. Despite these recommendations, screening rates for ID remain low among people with SUD due to numerous patient- and provider-level barriers to care [16–19]. For instance, only 8.5% of people who inject drugs (PWID) were ever tested for HIV and 7.7% were ever tested for HCV from 2010 to 2017 [16]. Additionally, among patients with SUD admitted inpatient for infection, just 14% received comprehensive screening for HIV, HCV, syphilis, chlamydia, and gonorrhea despite being seen by ID providers [20]. These findings highlight numerous missed opportunities for screening in various health care settings.

These screening challenges have led to calls for more integrated responses between ID specialists and SUD providers [21–23]. Integrated responses—such as offering on-site HIV and HCV testing at outpatient SUD programs [24] and using electronic health record smart phrases to prompt inpatient screenings [25]—have been implemented with demonstrated improvements in screening rates. In 2021, the Substance Abuse and Mental Health Services Administration released guidelines [26] encouraging medical staff members at substance use treatment programs to have a primary role in screening for hepatitis and HIV for all patients presenting to care. Moreover, licensing agencies for outpatient recovery programs have implemented mandates for co-located ID screening to decrease barriers to such screening [27].

Despite this guidance and the success of integrated screening interventions in other settings, the potential for inpatient SUD programs to serve as platforms for comprehensive ID screening, treatment, and prevention has not been fully explored. To address this knowledge gap, we evaluated the effectiveness of integrating systematic and comprehensive care for ID within an inpatient SUD treatment program.

## METHODS

### Design and Setting

We conducted an effectiveness study evaluating a comprehensive ID screening, prevention, and treatment intervention embedded within an inpatient substance use treatment unit. The inpatient withdrawal management and substance use recovery program was located at Penn Presbyterian Medical Center (PPMC), an affiliate hospital of the University of Pennsylvania Health System. This inpatient treatment program provided medically managed withdrawal and other treatment for adults ≥18 years old and was staffed by psychiatrists, registered nurses, and substance use counselors 24 hours per day, 7 days per week. The unit offered medications for opioid use disorder, medication treatment for other SUDs, and medication-free options. Patients could choose to participate in medically managed withdrawal services only or continue inpatient rehabilitation after initial withdrawal management. This project was reviewed and determined to qualify as quality improvement by the University of Pennsylvania's Institutional Review Board.

### Study Population

Patients were included in the study if they were admitted to the inpatient withdrawal management and substance use treatment program at PPMC during the study periods. Patients admitted during the intervention period (3 September 2022–30 June 2023) who were seen by the ID-trained nurse navigator were included in the intervention group to assess the effectiveness of the intervention. The intervention period was compared with a “usual care” period from 1 September 2021 through 28 June 2022. The observation period for the usual care group was chosen to account for potential seasonal patterns in unit admissions [28]. Only the first hospitalization in the study periods was included for patients with >1 admission to this unit.

### Usual Care

Prior to implementation of comprehensive ID testing, screening was completed at the discretion of the admitting psychiatrist. During this period, the PPMC had employed a default HCV screening and linkage program for all patients born between 1945 and 1965 admitted to any unit of the hospital [29]. Patients diagnosed with HCV viremia through this program were proactively engaged by the Hepatitis Linkage Team, counseled on the availability of curative treatment, and linked to an HCV-treating provider. While no additional opt-out testing, counseling, or follow-up of results was systematically offered, ID consultation could be requested by the psychiatrist for relevant care needs. All patients discharged from the unit were offered linkage to outpatient recovery services per practice standards during the usual care and intervention periods.

### Intervention

During the intervention phase, patients admitted to the unit met with an ID-trained nurse navigator (R.R.) during routine business hours on Mondays through Fridays. ID screening occurred in a staged fashion with HIV screening and viral hepatitis laboratory assays ordered at the time of admission. Consistent with Pennsylvania state law, all patients provided verbal consent for opt-out HIV screening at admission [30]. The nurse navigator subsequently met with each patient to review the HIV and viral hepatitis results and counsel the patient on indications for preventive interventions and/or treatment options. At this visit, the nurse navigator additionally obtained a sexual health and tuberculosis exposure history for each patient. Screening for STIs and LTBI was ordered when indicated by patient responses. Patients who completed ID screening within the prior 3 months did not repeat the screening assays. As comprehensive ID screening was tailored to personal history to minimize redundant or unnecessary testing, screening completion rates could vary across the ID evaluated.

### ID Screening Tests, Prevention, and Treatment

HIV screening was completed with a fourth-generation HIV antigen/antibody combination assay with confirmatory multispot [31]. Viral hepatitis screening included HAV total antibody, HBV surface antigen, HBV surface antibody, HBV core antibody (total), and HCV antibody with reflex to confirmatory HCV RNA. Screening assays for STIs included gonorrhea/chlamydia nucleic acid amplification test for urine, oropharyngeal swab, and rectal swab (if indicated by sexual health history) and rapid plasma reagin with reflex to treponemal antibody; trichomonas screening via urine polymerase chain reaction assay was completed for sexually active women only [14]. An interferon gamma release assay for LTBI was completed for patients without prior positive LTBI test result or history of TB treatment.

Preventive interventions systematically offered during the intervention period included vaccinations for HAV and HBV and preexposure prophylaxis (PrEP) for HIV. Patients who screened positive for any infection were evaluated by an ID physician for further care. Treatment was offered to all patients with HIV, HCV, and STIs while admitted. For patients diagnosed with HBV, HCV, HIV, or LTBI, outpatient follow-up was scheduled by the nurse navigator prior to hospital discharge to ensure longitudinal care. Patients who expressed interest in PrEP were scheduled by the nurse navigator with a PrEP-prescribing provider prior to hospital discharge. Patients in need of viral hepatitis vaccine series completion were counseled on timing of future vaccine need and availability of vaccine at primary care, commercial pharmacies, or local health centers. Laboratory testing, vaccines, and medications ordered as part of this intervention were part of routine clinical care; associated costs were included within the admission and/or billed to the patient's insurer.

### Data Collection

Medical record reviews were performed to abstract demographic data, including age, gender, race, and ethnicity at the time of index hospitalization. As type of substance consumed is not reported in structured text fields, the history and physical document was reviewed for documentation of patient-reported consumption of substances (ie, cocaine, opioids, alcohol, or other) within the past 12 months. Documentation of patient-reported injection drug use within the past 12 months was also abstracted. Screening ID laboratory results completed during the index hospitalization were collected. Dispensation of HAV or HBV vaccination or treatment for HBV, HCV, HIV, or STIs during the index admission was abstracted. Linkage to outpatient ID follow-up care was confirmed if the nurse navigator or discharging physician documented the appointment in a progress note or discharge summary. For patients with HCV viremia, additional abstraction was performed to determine prescription and dispensation of indicated therapy for HCV following discharge. Hospital characteristics, including length of stay and patient-directed discharge, were obtained from the discharge summary.

### Study and Outcome Definitions

Effectiveness of the intervention was defined as the proportion of patients completing any ID screening laboratory tests. Prevalence of ID treatment or prevention needs was assessed, as was patient uptake of any ID treatment or preventive services during the patient's index hospitalization.

Preventive care needs were defined for HAV, HBV, and HIV prevention. People with nonreactive HAV antibody and nonimmune HBV triple serologies (ie, nonreactive surface antibody, nonreactive core IgG antibody, and negative surface antigen) were considered eligible for vaccination. As prior STIs, injection drug use, and sexual risk exposure history may be underreported [32, 33], all patients who tested negative for HIV were presumed to be potential PrEP candidates; however, only patients who expressed interest in PrEP were linked to care during the intervention period. Any prevention provided was defined as at least 1 HAV or HBV vaccine administered during the index hospitalization or the patient meeting with the PrEP navigator during hospitalization.

Treatment or evaluation indications and initiation were defined for HBV, HCV, HIV, LTBI, and STIs. Patients with a reactive HBV surface antigen were evaluated by the ID physician to determine treatment need. All patients with HCV viremia were considered eligible for direct-acting antiviral treatment. All patients with untreated HIV were considered eligible for initiation of antiretroviral therapy. Patients with a positive interferon gamma release assay result completed an evaluation with the ID physician to review TB infection and treatment history and complete symptom review and chest x-ray; LTBI treatment was initiated at the discretion of the ID physician. Treatment for gonorrhea, chlamydia, or trichomonas was prescribed after review of the positive test result with the patient. Patients with a reactive rapid plasma reagin and positive treponemal antibody had treatment history confirmed through query of the local health department registry; patients in need of syphilis treatment were evaluated by the ID physician prior to initiation of treatment.

### Statistical Analysis

Descriptive statistics were used to compare proportions of patients with completion of ID screening, identification of intervention need, and delivery of treatment or preventive care in the usual care vs intervention groups. Patients who initiated an antimicrobial course for an STI or antiviral therapy during the admission and/or had a referral to outpatient care for HBV, HCV, HIV, or LTBI were considered “treated” in the analyses. A Kruskal-Wallis test and χ^2^ or Fisher exact test were used for comparison of continuous and categorical variables, respectively.

To define overall uptake of the intervention, composite binary definitions were used to compare completion of any ID screening tests, any indication for ID treatment or prevention, and delivery of any ID treatment or prevention. The following comparisons were performed: any ID screening completion, indication for any ID treatment/preventive care, and delivery of any ID treatment/preventive care. Participants with multiple screening tests completed, administered treatments, or preventive interventions contributed only once to each comparison. Additional exploratory analyses evaluated ID screening and (1) preventive care for HAV, HBV, and HIV and (2) treatment indication and delivery for HBV, HCV, HIV, LTBI, gonorrhea, chlamydia, syphilis, or trichomonas. All analyses were performed in STATA/BE software version 18.0 (StataCorp). Statistical significance was set a priori at *P* < .05.

## RESULTS

Of the 515 unique patients admitted to the substance use treatment unit, 261 (50.7%) were admitted during the usual care period and 254 (49.3%) during the intervention period. Of the 254 patients admitted during the intervention period, 207 were approached by the nurse navigator and included in the effectiveness analysis. The 47 patients excluded from the analysis were unable to meet with the nurse navigator due to hospitalizations occurring exclusively over a weekend or holiday. Patient demographics were similar across the usual care and intervention groups (Table 1). Reported substance usage was notable for greater alcohol (*P* < .001) and other substance use (*P* < .001) in the intervention group. A greater prevalence of injection drug use and opioid use was observed in the intervention group; however, this did not meet statistical significance.

**Table 1.** Baseline Characteristics of Patients by Infectious Disease Screening Period

Characteristic	Usual Care (n = 261)	Intervention (n = 207)	*P* Value
Age, y, median (IQR)	48 (39–56)	50.0 (39–58)	.11
Race			.15
Black	163 (62.5)	144 (69.6)	
White	79 (30.3)	55 (26.6)	
Other	19 (7.3)	8 (3.9)	
Hispanic ethnicity	12 (4.3)	8 (3.9)	.83
Gender			.83
Cisgender male	170 (65.1)	137 (66.2)	
Cisgender female	89 (34.1)	67 (32.4)	
Transgender female	2 (0.8)	3 (1.4)	
Injection drug use	45 (17.2)	45 (21.7)	.22
Reported substance use^a^			
Opioid	112 (42.9)	100 (48.3)	.24
Cocaine	169 (65.0)	126 (60.9)	.36
Alcohol	156 (59.8)	89 (43.0)	<.001
Other substance	146 (55.9)	63 (30.4)	<.001
Hospitalization			
Length of stay, d, median (IQR)	12 (5–24)	16 (5–29)	.21
Patient-directed discharge	69 (26.4)	48 (23.2)	.42

Data are presented as No. (%) unless noted otherwise.

^a^Reported substance use categories are not mutually exclusive.

### Composite ID Screening, Prevention, and Treatment

**Figure 1.**
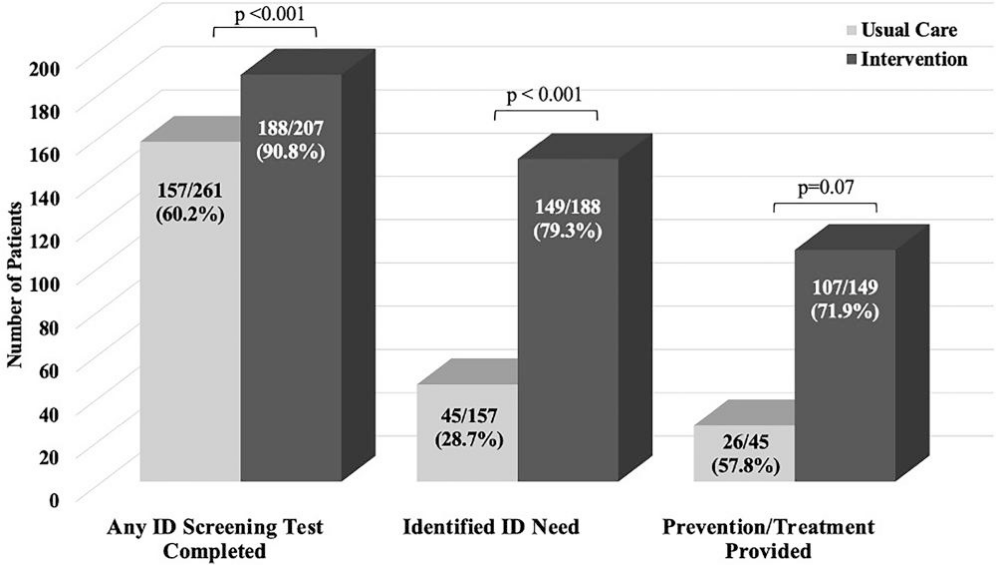
Impact of embedded comprehensive infectious disease (ID) screening on screening completion, ID identification, and delivery of treatment and/or prevention. A bar chart compares the outcomes between usual care and intervention in an ID screening study among patients with substance use disorder. The x-axis has 3 categories: any ID screening test completed, the identified ID need, and the prevention/treatment provided. For each category, the usual care group is shown in light gray and the intervention group in dark gray. For any ID screening test completed, 157 of 261 (60.2%) completed screening under usual care as compared with 188 of 207 (90.8%) in the intervention (*P* < .001). For identified ID need, 45 of 157 (28.7%) under usual care had an identified ID need as compared with 149 of 188 (79.3%) in the intervention (*P* < .001). Finally, for prevention/treatment provided, 26 of 45 (57.8%) received care in the usual group as compared with 107 of 149 (71.9%) in the intervention group (*P* = .07). The chart shows significantly improved outcomes in the intervention group.

Completion of any ID screening tests and identification of treatment or prevention needs were significantly higher during the intervention period (Figure 1). Any ID screening was completed for 60.2% and 90.8% of patients in the usual care and intervention groups, respectively (*P* < .001). The prevalence of any identified treatment or prevention needs significantly increased from 28.7% to 79.3% in the intervention group (*P* < .001). Provision of any ID treatment or preventive service during patients’ inpatient stays was delivered to 4 times more patients in the intervention group; however, due to the lower identification of treatment/prevention needs in the usual care period, the proportion of people receiving any ID prevention/treatment did not meet statistical significance (57.8% vs 71.9%, *P* = .07).

### Screening and Prevention for HAV, HBV, and HIV

The greatest increase in ID screening during the intervention was observed for HAV (6.1% vs 90.3%, *P* < .001) and HBV (8.8% vs 91.3%, *P* < .001; Table 2). HAV and HBV nonimmunity was common between the groups, with 68.8% and 45.5% being nonimmune for HAV and 34.8% and 48.6% being nonimmune for HBV in the usual care and intervention groups, respectively. During the intervention phase, HAV and HBV vaccination significantly increased, with 50 vaccination series initiated for HAV and 60 for HBV as compared with no vaccinations delivered for either HAV or HBV in the usual care period.

**Table 2.** Completion of Hepatitis A, Hepatitis B, and HIV Screening and Delivery of Preventive Interventions Before and After Implementation of a Comprehensive Embedded Infectious Disease Screening Program

Screening and Prevention	Usual Care (n = 261)	Intervention (n = 207)	*P* Value
Hepatitis A virus			
Completed screening	16/261 (6.1)	187/207 (90.3)	<.001
Nonimmune	11/16 (68.8)	82/187(45.5)	.07
Vaccine administered	0/11 (0.00)	50/82 (58.0)	<.001
Hepatitis B virus			
Completed screening	23/261 (8.8)	189/207 (91.3)	<.001
Non-immune	8/26 (34.8)	85/189 (48.6)	.21
Vaccine administered	0/8 (0.0)	60/85 (71.0)	<.001
HIV			
Completed screening	62/261 (23.8)	159/207 (76.8)	<.001
Negative HIV assay	44/62 (71.0)	128/159 (80.5)	.11
Linked to PrEP^a^	0/44 (0.0)	7/128 (5.2)	.12
Started on PrEP^b^	…	3/7 (42.9)	…

Data are presented as No. (%) unless noted otherwise.

Abbreviation: PrEP, preexposure prophylaxis.

^a^Intervention needed for HIV includes those who were offered PrEP by their providers, but it may not be all-inclusive as other participants may have had PrEP indications but were not offered PrEP.

^b^Started oral tenofovir disoproxil fumarate/emtricitabine, tenofovir alafenamide/emtricitabine, or long-acting injectable cabotegravir during admission.

HIV screening increased from 23.8% to 76.8% during the intervention period. No new HIV diagnoses were identified; however, in both study periods, all patients with HIV admitted without antiretroviral therapy resumed their last known regimen and were linked to outpatient HIV care per the hospital's standard of care. PrEP was not offered or provided to any patient in the usual care period. During the intervention, PrEP provision significantly increased (*P* < .001), but only 3 patients eligible for PrEP initiated therapy during admission.

### Screening and Treatment of HBV, HCV, LTBI, and STIs

One patient in the usual care period and 2 patients in the intervention were HBV surface antigen positive. In the intervention phase, 1 of these patients self-discharged before further ID evaluation or linkage could be completed. The remaining 2 patients initiated HBV-active therapy during their admission, following ID physician evaluation.

HCV screening was high in both phases (49.0% vs 91.6%) due to an existing default HCV screening program for people born from 1945 to 1965 at the hospital at the time of the usual care period. HCV viremia was found in 15 and 19 patients in the usual care and intervention groups, respectively. For patients who were HCV viremic, linkage to care was achieved in 67.0% of usual care and 100% of intervention. Notably, 7 (36.8%) patients who were viremic in the intervention phase started direct-acting antiviral therapy within 2 weeks of noted viremia, including 3 who started it while still admitted in the unit.

Screening within the usual care group was lowest for LTBI (1.9%) and improved significantly to 67.6% (*P* < .001) after implementation of the intervention (Table 3). Only 1 patient tested positive in the usual care period and 14 tested positive in the intervention period. However, the majority of positive results in the intervention phase (8/14, 57.1%) were ultimately deemed to be false positives or reflective of previously treated LTBI. One patient in the intervention group with LTBI was prescribed rifampin while admitted.

**Table 3.** Infectious Disease Screening and Treatment Outcomes Before and After Implementation of the Comprehensive Embedded Infectious Disease Screening Program

Screening and Treatment	Usual Care (n = 261)	Intervention (n = 207)	*P* Value
Hepatitis B			
Completed screening	23/261 (8.8)	189/207 (91.3)	<.001
Treatment indicated	1/23 (4.3)	2/189 (1.1)	.21
Referred to treatment	1/1 (100.0)	1/2 (50.0)	.39
Hepatitis C			
Completed screening	129/261 (49.4)	190/207 (91.8)	<.001
Hepatitis C antibody positive	37/129 (28.7)	44/190 (23.2)	.27
Hepatitis C viremia	15/37 (41.0)	19/44 (43.0)	.81
Referred to treatment	10/15 (67.0)	19/19 (100.0)	.006
Started DAA within 2-wk discharge	2/10 (20.0)	7/19 (36.8)	.35
HIV			
Completed screening	62/261 (23.8)	159/207 (76.8)	<.001
HIV test positive	3/62 (6.4)	13/159 (8.7)	.62
Referred to treatment	3/3 (100)	13/13 (100)	>.99
Latent tuberculosis			
Completed screening	5/261 (1.9)	140/207 (67.6)	<.001
Positive IGRA	1/5 (20.0)	14/140 (10.0)	.49
Evaluation completed^a^	0/1 (0.0)	12/14 (80.0)	.07
Gonorrhea/chlamydia			
Completed screening	39/261 (14.9)	124/207 (59.9)	<.001
Treatment indicated	0/39 (0.0)	2/124 (1.7)	.42
Treatment initiated	0/0 (0.0)	2/2 (100)	<.001
Syphilis			
Completed screening	48/261 (18.4)	141/207 (68.1)	<.001
RPR and treponemal antibody positive	8/48 (16.7)	13/141 (9.2)	.16
Evaluation completed^a^	5/8 (62.0)	11/13 (85.0)	.25
Trichomonas^b^			
Completed screening	25/89 (27.5)	38/67 (56.7)	<.001
Treatment indicated	18/25 (72.0)	15/38 (39.0)	.011
Treatment initiated	13/18 (72.0)	15/15 (100)	.027

Data are presented as No. (%) unless noted otherwise.

Abbreviations: DAA, direct-acting antiviral; IGRA, interferon gamma release assay; RPR, rapid plasma reagin.

^a^Evaluation completed by infectious disease physician and included physical examination, symptom evaluation, and consideration of prior results and treatment history. Not all patients evaluated required treatment.

^b^Screening for trichomonas completed for cisgender females at high risk for infection (eg, multiple sex partners, transactional sex, drug misuse, or a history of sexually transmitted infection or incarceration) per 2021 sexually transmitted infection guidelines of the Centers for Disease Control and Prevention [14].

Screening for STIs increased significantly for gonorrhea, chlamydia, syphilis, and trichomonas in the intervention period (Table 3). The prevalence of test positivity for each infection was similar despite the increased frequency of screening in the intervention group. Notably, the prevalence of trichomoniasis was high: 72% in usual care and 39% in the intervention period. Trichomonas treatment initiation for those testing positive was 100% in the intervention period as compared with 72% in usual care.

## DISCUSSION

To our knowledge, this is the first study exploring comprehensive embedded ID screening, treatment, and prevention during inpatient substance use treatment. Completion of ID screening substantially increased with the intervention, with the greatest increases observed for HAV, HBV, and LTBI screening. Comprehensive ID screening identified more treatment and prevention needs, with 79.3% of patients having at least 1 identified need during the intervention as compared with 28.7% of patients who received usual care. Importantly, patients offered comprehensive ID services were treated or offered prevention more frequently during the intervention phase as compared with the usual care phase.

Our data reinforce previous findings that embedding ID screening services into nontraditional settings can improve screening rates. In an outpatient SUD program, Brooks et al found that on-site HIV and HCV testing improved completion of screening from 13% to 90% for HIV and from 4% to 90% for HCV [24]. A similarly bundled ID screening intervention improved completion of screening among PWID admitted with serious injection-related infections for all conditions, with statically significant increases in screening for STIs [25]. Our intervention led to statistically significant improvement in all ID screening laboratory tests ordered. Taken together, our findings support adding inpatient substance use treatment programs as additional venues that may serve as engagement opportunities in this hard-to-reach patient population.

Our intervention was particularly impactful at improving delivery of treatment and preventive services. Although screening improved in the previously cited study with an inpatient bundled ID screening program for PWID, delivery of preventive services, including vaccinations and PrEP, remained low: in particular, vaccination remained stable at 8% for HAV and HBV despite the intervention [25]. In contrast, our intervention led to substantial improvements in the delivery of treatment and preventive services, with HAV and HBV vaccination achieving 58% and 71% administration, respectively. Moreover, systematic trichomonas screening, which was not performed in these prior studies, resulted in meaningful improvements in treatment completion during the intervention (100%) relative to usual care (72%). Therefore, our findings argue for prioritization of viral hepatitis screening to facilitate vaccination, as well as trichomonas screening and treatment, given the high prevalence if ID resources are limited.

While our program demonstrated modest improvements in PrEP referral and initiation, uptake of PrEP in our patients remained low. Prior work has demonstrated that PWID have a high willingness to use PrEP, yet PrEP uptake did not exceed 3% due to multiple factors, including low perceived risk, medication toxicity concerns, and stigma [34]. Further work is needed to define optimal interventions to improve PrEP uptake in people with SUD.

Our study has several potential limitations. Our intervention was performed at a single center within 1 SUD treatment unit and was evaluated in an effectiveness analysis. Given the nonrandomized sequential design, the potential of time-varying confounders cannot be excluded. During our study period, rates of STIs and fentanyl use increased in Philadelphia [35]. Increasing fentanyl use has led to more frequent injection and needle sharing [36]; thus, increasing detection for ID in the intervention group may have been affected by these trends. Despite this potential for bias, this simply highlights the importance of this intervention. Finally, the generalizability of our findings may be limited to settings with similar clinical resources. Our nurse navigator was a 0.5 full-time equivalent role funded by the Philadelphia Department of Public Health for development, implementation, and evaluation of the intervention. Face-to-face time with patients ranged from 5 to 60 minutes/patient over the course of the admission. With this limited additional time required to complete ID result review, counseling treatment, and/or vaccination, this comprehensive embedded ID screening approach can be feasible without requiring dedicated staff.

Our study has several potential strengths. Cited barriers to provision of ID treatment or prevention in nontraditional settings include competing priorities during the admission, patient refusal, or self-directed discharge [25]. Thus, our intervention may have created solutions to several of these potential barriers. For example, numerous studies demonstrate that patients who have their addiction sufficiently treated during admissions have better outcomes and are more likely to complete treatments [37, 38]. As such, our intervention may have led to meaningful improvements in delivery of treatment and preventive services because patients’ substance withdrawal symptoms were being appropriately managed, making patients less likely to leave prior to administration. Additionally, the utilization of a dedicated and ID-trained nurse navigator may have mitigated the effects of competing demands as a barrier to delivery of ID treatment and prevention. While further study is needed to identify specific facilitators, our work highlights the missed treatment and prevention opportunities that currently exist in these settings and the potential for bundled screening, treatment, and preventive services to improve wholistic ID care in patients with SUD.

In conclusion, our study demonstrates that comprehensive screening for ID is effective and widely accepted by patients during inpatient substance use treatment admissions. Embedding ID screening, treatment, and prevention services into inpatient substance use treatment results in timely identification of ID and facilitates prompt delivery of preventive services, treatment of infections, and linkage to care. This comprehensive approach can serve as an opportunity for engagement and care in a patient population that experiences stigma and other barriers to care.

## Notes

***Author contributions*.** All listed authors approved this manuscript and have contributed meaningfully to this work.

***Patient consent statement*.** This project was reviewed and determined to qualify as quality improvement by the University of Pennsylvania's Institutional Review Board. Patient consent was not required for this study.

***Financial support***. This work was supported in part by the Department of Public Health, City of Philadelphia (principal investigator, J. T.; contract 21220506130909); the National Institute of Diabetes and Digestive and Kidney Diseases (principal investigator, J. T.; K08-DK132977); and the Agency for Healthcare Research and Quality (principal investigator, Peter Groeneveld; T32HS026116).
